# Risk, resilience, psychological distress, and anxiety at the beginning of the COVID‐19 pandemic in Germany

**DOI:** 10.1002/brb3.1745

**Published:** 2020-07-07

**Authors:** Moritz Bruno Petzold, Antonia Bendau, Jens Plag, Lena Pyrkosch, Lea Mascarell Maricic, Felix Betzler, Janina Rogoll, Julia Große, Andreas Ströhle

**Affiliations:** ^1^ Department of Psychiatry and Psychotherapy Charité – Universitätsmedizin Berlin, corporate member of Freie Universität Berlin, Humboldt‐Universität zu Berlin, and Berlin Institute of Health Berlin Germany

**Keywords:** adjustment disorders, anxiety/anxiety disorders, coping, corona, depression

## Abstract

**Background:**

The current COVID‐19 pandemic comes with multiple psychological stressors due to health‐related, social, economic, and individual consequences and may cause psychological distress. The aim of this study was to screen the population in Germany for negative impact on mental health in the current COVID‐19 pandemic and to analyze possible risk and protective factors.

**Methods:**

A total of 6,509 people took part in an online survey in Germany from 27 March to 6 April. The questionnaire included demographic information and ascertained psychological distress, anxiety and depressive symptoms, and risk and protective factors.

**Results:**

In our sample, over 50% expressed suffering from anxiety and psychological distress regarding the COVID‐19 pandemic. Participants spent several hours per day thinking about COVID‐19 (*M = *4.45). Psychological and social determinants showed stronger associations with anxiety regarding COVID‐19 than experiences with the disease.

**Conclusions:**

The current COVID‐19 pandemic does cause psychological distress, anxiety, and depression for large proportions of the general population. Strategies such as maintaining a healthy lifestyle and social contacts, acceptance of anxiety and negative emotions, fostering self‐efficacy, and information on where to get medical treatment if needed, seem of help, while substance abuse and suppression of anxiety and negative emotions seem to be associated with more psychological burden.

## Graphical abstract

The aim of this study was to screen the population in Germany for negative impact on mental health in the current COVID‐19 pandemic and to analyze possible risk and protective factors. A total of 6,509 people took part in an online survey. Over 50% expressed suffering from anxiety and psychological distress regarding the COVID‐19 pandemic. Participants spent several hours per day thinking about COVID‐19. Psychological and social determinants showed stronger associations with anxiety regarding COVID‐19 than experiences with the disease.

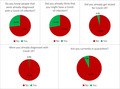

## 1 INTRODUCTION

The new virus SARS‐CoV‐2 has now rapidly spread to nearly all countries over the world, and the World Health Organization (WHO) declared an international pandemic in March 2020 (Ghebreyesus, 2020). The pandemic comes with a large number of potential stressors that might cause psychological distress and mental health burden (Inter‐Agency Standing Committee, 2020). Potential stressors related to the virus might be the fear of an infection with COVID‐19 and the consequences for oneself or loved ones. The taken measures that aim to slow down the spreading of the virus also come with lots of stressors such as social isolation, economic consequences, and uncertainty about the future (Inter‐Agency Standing Committee, 2020). Therefore, an increase in psychological distress and negative consequences for the mental health of large populations worldwide can be assumed. In a rapid developing situation with a pandemic of a scale that was not known in the last 50 years, substantial research on the psychological consequences of the pandemic is lacking. First studies provide evidence regarding psychological distress in the context of the COVID‐19 pandemic. An online survey in the general population in China (Wang, Pan, Wan, Tan, Xu, Ho, et al., 2020) showed that more than half of the participants rated the psychological impact of the events as moderate‐to‐severe and 16.5% reported depressive and 28.8% anxiety symptoms of moderate‐to‐severe intensity during the initial stage of the pandemic. These proportions seemed to be relatively stable—a second survey 4 weeks later showed no significant reduction in those symptoms (Wang, Pan, Wan, Tan, Xu, McIntyre, et al., 2020). Another study from China showed a lower prevalence of symptoms of psychological distress in Chinese workforce during the COVID‐19 outbreak (Tan, Chew, et al., 2020; Tan, Hao, et al., 2020), and particularly, individuals with preexisting (mental) health issues seem to suffer from psychological strain in the context of the pandemic (Hao et al., 2020).

Studies that focused on the psychological consequences of previous epidemics or pandemics showed that these were associated with substantial psychological distress and mental health problems, for example, during the Ebola epidemic 2014 (Greenberg, Wessely, & Wykes, 2015; Mohammed et al., 2015) or the SARS outbreak in 2003 (Maunder et al., 2006).

### 1.1 Pandemic situation in Germany

**Figure 1:**
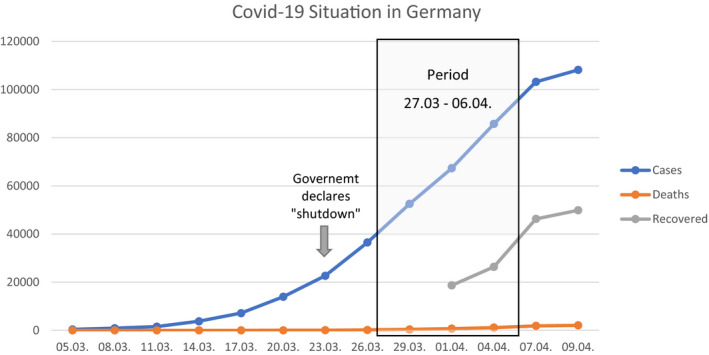
COVID‐19 situation during recruitment. ^a^Data from Robert Koch Institut (RKI, 2020)

The first case in Germany was detected in January 2020 (Bayrisches Staatsministerium für Gesundheit und Pflege, 2020), and case numbers have been rising afterward (see Figure 1). In parallel, stepwise more rules appeared to inhibit a further exponential growth of the infection numbers, for example, the closure of all educational, cultural and gastronomical institutions, and a reduction in retail and service sectors (Bundesgesundheitsministerium, 2020). Since 23 March, throughout Germany, more rigorous national rules became effective, including further closures of institutions and restrictions of physical contact and staying outside. To our knowledge, there is no published research on factors of psychological distress in the general population in Germany during the current pandemic. Hence, the aim of the present study was to assess psychological distress, anxiety, and depression with regard to the COVID‐19 pandemic and to analyze possible risk and protective factors.

## 2 METHODS

### 2.1 Design

This is a cross‐sectional observational study using a convenience sample of the general population in Germany via online survey, approved by the ethics committee of Charité Universitätsmedizin Berlin (EA1/071/20) and registered on ClinicalTrials.gov (NCT04331106).

### 2.2 Recruitment

To survey the psychological dimension of the COVID‐19 pandemic, an online self‐report questionnaire via *SoSci Survey* was used. Data collection started 27 March 2020, when in Germany, 42,288 cases of infection and 253 deaths attributed to COVID‐19 were reported (Robert Koch Institut, 2020). The end of the first wave of data collection was 10 days later: 6 April 2020, when in Germany 95,391 cases and 1,434 deaths were reported (Robert Koch Institut, 2020). Recruitment was primarily done via social media and the website of the Charité. Completing the entire survey required 10–15 min. The present paper only examines cross‐sectional data of the first wave. Further longitudinal measurements will be carried out. All participants gave informed consent prior to participation. Figure 1 shows the COVID‐19 situation in Germany during recruitment period regarding cases of infection, death, and recovery.

### 2.3 Eligibility criteria

Except the minimum age of 18 years, residence in Germany, and the ability to complete the questionnaire in German, there were no other inclusion or exclusion criteria.

### 2.4 Assessment

The online questionnaire contained demographic information and the experiences with the virus (e.g., being in quarantine, tested or diagnosed for the coronavirus). Additionally, the subjective risk of being infected within the next month was rated from 0% to 100% and the daily average amount of hours spent thinking about COVID‐19 was recorded.

To screen for general anxiety and depressive symptoms, the ultra‐brief screening scale of the Patient Health Questionnaire‐4 (PHQ‐4) (Löwe et al., 2010) was used. The intensity of four items describing major anxiety/depressive symptoms was rated on a 4‐point scale from 0 (“not at all”) to 3 (“nearly every day”). The PHQ‐4 can be examined as a total score or be divided into an anxiety (GAD‐2) and a depression subscale (PHQ‐2).

To assess selected aspects of anxiety regarding COVID‐19, nine items were included (e.g., the fear of being infected and the fear of social or economic consequences). All statements were rated on a 6‐point Likert scale, ranging from 1 (“not true at all”) to 6 (“totally true”). Additionally, a modified version of the validated DSM‐5 Severity‐Measure‐For‐Specific‐Phobia‐Adult‐Scale (Beesdo‐Baum et al., 2012) was used to ascertain the extent of anxiety symptoms caused by the pandemic. The scale consists of 10 items, rated on a 5‐point Likert scale from 0 (“never”) to 4 (“all the time”).

The questionnaire inquired eight items regarding protective factors in dealing with the pandemic (e.g., self‐efficacy in general, social self‐efficacy) and five items targeting risk factors (e.g., suppression, substance use). Protective and risk factors were adapted from the recommendations on coping with psychological distress in the pandemic of the Inter‐Agency Standing Committee (IASC) of the United Nations (UN) (Inter‐Agency Standing Committee, 2020). Items were rated on a 6‐point Likert scale. All questions were administered in German.

### 2.5 Data analysis

The questionnaire consisted of eight pages. We included only participants who completed at least page 4 (*N* = 6,509). 93.6% of the participants (*N* = 5,721) completed all pages. Average percentage of missing data on item level was 2.1% (range: 0.0–7.1). Missing data were handled by casewise deletion. All analyses were carried out using IBM SPSS Statistics Version 25. Significance level was set to .05 (two‐tailed). For the analysis, descriptive statistics, Pearson’s and Spearman’s correlations, and *t* tests for independent samples were used.

## 3 RESULTS

### 3.1 Sample characteristics

70.1% of the participants were female (*N* = 4,563), 29.0% male (*N* = 1,887), and 0.9% identified as diverse (*N* = 59). Mean age was 36.2 years (*SD* = 11.65, range 18–99). 37.6% reported to have children (*N* = 37.6%). 15.1% had a secondary school degree (*N = *985), 32.4% had a higher education entrance qualification (*N* = 2,109), and 50.0% had a university degree (*N = *3,254). 16.7% of the participants reported to work in a medical context (*N = *1,084). 10.7% of the participants suffered from a severe physical illness (*N* = 695). The participants lived in a household with 2.54 persons on average (including themselves).

### 3.2 Experiences with COVID‐19

**Figure 2:**
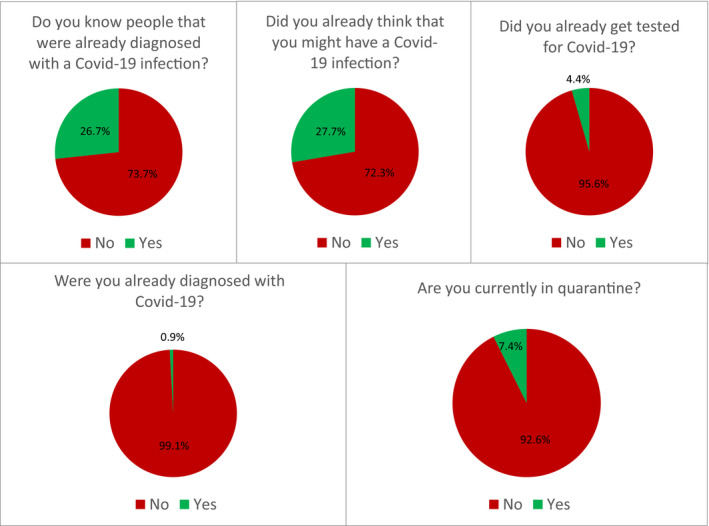
Experiences with COVID‐19 (*N* = 6,509)

Figure 2 shows the experiences of the participants with COVID‐19. About one third of the participants knew someone diagnosed with COVID‐19 or already suspected themselves to be infected. About 7% were currently under quarantine, and <5% had been tested for COVID‐19. About 1% of the sample had been diagnosed with COVID‐19.

### 3.3 Risk perception and contact

Average rating of the risk of being infected with COVID‐19 within the next month was 38.3% (*SD* = 25.26, range: 0–100). Most participants rated the risk with 50% (21.8%, *N* = 1,422). The lowest 25% of the sample ranked it as 20.0% or lower. Median of risk perception was 40.0%. The highest 25% ranked the risk at least as 50%. Average rating of the risk of being infected with influenza (“flu”) was 18.2% (*SD* = 19.89, range: 0–100) and the median 10.0%. Most participants rated the risk with 10.0% (20.6%, *N* = 1,341). Women evaluated both risks higher than men (COVID‐19: *M* = 40.17%; *SD* = 0.37 vs. *M* = 33.93%; *SD* = 0.58; *p* < .001; influenza: *M* = 18.92%; *SD* = 0.30 vs. *M* = 16.60%; *SD* = 0.43, *p* < .001).

25.7% (*N = *1,673) of the participants did not have any contact with persons closer than one‐meter distance outside of their household during the last week. 40.2% (*N = *2,916) reported contact with 1–3 persons and 24.3% (*N* = 1578) with 4–10 persons, while 9.9% (*N* = 642) reported contact with 10 or more persons. There were no significant gender differences in the amount of contact.

### 3.4 Hours spent thinking about COVID‐19

On average, the participants thought about COVID‐19 for 4.45 hr/day (*SD* = 3.80, range from 0 to 24). 25% of the participants thought <2 hr, while 25% thought 6 hr or more per day about COVID‐19. 10% reported to think more than 10 hr/day about COVID‐19. Women spent significantly more hours per day thinking than men (*M* = 4.57; *SD* = 3.82 vs. *M* = 4.15, *SD* = 3.75; *p* < .00).

Participants who spent 2 hr or more thinking about COVID‐19 differed significantly from participants who thought <2 hr about COVID‐19. The former showed higher scores in the PHQ‐4 (*M* = 4.6, *SD* = 3.2 vs. *M* = 2.6, *SD* = 2.5; *p* < .001) and in the phobia questionnaire (*M* = 1.2, *SD* = 0.7 vs. *M* = 0.6, *SD* = 0.4; *p* < .001).

### 3.5 Anxiety regarding COVID‐19

**Figure 3:**
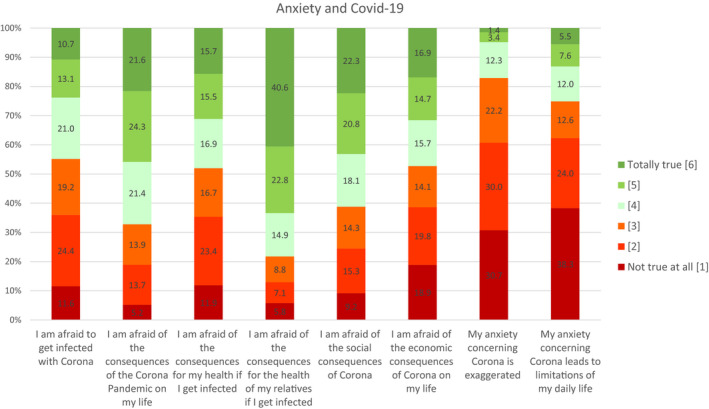
Anxiety regarding COVID‐19

Figure 3 shows the distribution of answers to the questions about anxiety regarding COVID‐19.

44.8% of the participants agreed to be afraid to get infected with COVID‐19. 67.7% were afraid of the consequences of COVID‐19 for their personal life. 48.1% reported to be afraid of the consequences for their personal health if getting infected. 78.3% reported to be afraid of the consequences for the health of their relatives. 61.2% were afraid of the social consequences, while 47.3% reported to be afraid of the economic consequences on their life. 17.1% of the participants stated that their concern about COVID‐19 was exaggerated, and 25.1% stated that their anxiety would lead to limitations in their daily life. Women showed higher rates of anxiety in almost every item. The strongest differences compared to men could be found in anxiety of experiencing general (*M* = 4.23 vs. *M* = 3.81) and social consequences (*M* = 4.06 vs. *M* = 3.61) due to COVID‐19.

### 3.6 Associations with COVID‐19 anxiety

Table 1 shows correlations of demographics, experiences with corona, and protective and risk factors with selected aspects of anxiety regarding COVID‐19. People of higher age (*r* = .012, *p* < .001) stated to think more hours per day about COVID‐19. People suffering from a severe physical illness reported less hours (*r* = −.08, *p <* .001). Experiences with COVID‐19 showed some small statistically significant but no meaningful correlations with anxiety regarding COVID‐19. All different forms of self‐efficacy showed significant negative correlations with all aspects of COVID‐19 anxiety (range from *r =* −.08 to −.46). Normalization, social contacts, and knowledge where to get medical treatment showed significant negative correlations ranging from *r =* −.07 to *r* = −.24.

**Table 1:** Associations of demographics, experiences with corona, and protective and risk factors with anxiety

	I am afraid of the consequences of the corona pandemic on my life	My anxiety concerning corona is exaggerated	My anxiety concerning corona leads to limitations in my daily life	Hours thinking about corona per day
Demographics				
Age	−.018	.007	.028*	**.119** ***
Having children	−.030*	.010	−.012	−.065***
Working in medical context	.051**	.060***	.070**	−.044***
Preexisting severe physical illness	−.064**	−.040***	−**.122** ***	−.080***
Number of people in household	−.007	−.013	−.001	−.011
Experiences with corona				
Being in quarantine	.014	.033**	.071***	.031***
Knowing someone diagnosed	−.007	−.003	−.020	.008
Suspected to be infected	.048***	.080***	.075***	.050***
Having been tested	−.012	−.007	.006	.032
Having been diagnosed	−.020	−.014	.001	.010
Recovered	−.035	.011	−.010	.016
Protective factors				
Self‐efficacy general	−**.414** ***	−**.198** ***	−**.457** ***	−**.292** ***
Self‐efficacy health	−**.221** ***	−**.179** ***	−**.319** ***	−**.222** ***
Self‐efficacy social	−**.376** ***	−**.108** ***	−**.322** ***	−**.186** ***
Self‐efficacy economic	−**.363** ***	−**.078** ***	−**.220** ***	−**.134** ***
Normalization	−**.191** ***	−**.236** ***	−**.323** ***	−**.195** ***
Social contacts	−**.168** ***	−**.073** ***	−**.206** ***	−**.091** ***
Know where to get medical treatment	−**.135** ***	−**.072** ***	−**.159** ***	−**.095** ***
Know where to get psychosocial treatment	−.094***	−.043***	−.097***	−.081***
Risk factors				
Suppression	**.339** ***	**.306** ***	**.423** ***	**.232** ***
Reduced physical activity	**.170** **	**.097** ***	**.192** ***	**.125** ***
Reduced healthy diet	**.179** ***	**.105** ***	**.198** ***	**.149** ***
More substance use	**.143** ***	.077***	**.173** ***	**.143** ***

* Significant at .05 level;

** Significant at .01 level;

*** Significant at .001 level; and bold values represent significant values of a size of at least .1

### 3.7 Specific COVID‐19 phobia symptoms

The overall score of the modified Specific‐Phobia Scale was 10.15 (*SD* = 6.95), with women showing significantly higher scores than men (*M* = 10.67, *SD* = 6.94 vs. *M* = 8.88, *SD* = 6.78; *p* > .001).

### 3.8 Depressive and anxiety symptoms

The participants showed an average PHQ‐4 Score of 4.15 (*SD = *3.19, range 0–12). 25% of the participants showed a score of at least 6, while 10% of them showed a score of at least 9. Women showed a significantly higher PHQ‐4 Score (indicating more depressive and anxious symptomatology) than men (*M* = 4.4 vs. *M = *3.5).

The participants showed an average PHQ‐2 Score of 2.11 (*SD* = 1.70, range 0–6). 25% of the sample showed a score of at least 3 and 10% a score of at least 5. The average GAD‐2 Score was 2.03 (*SD* = 1.76, range 0–6). 25% of the participants showed a score of at least 3, while 10% showed a score of at least 5.

## 4 DISCUSSION

In this study, we wanted to explore how the current COVID‐19 pandemic is connected to a psychological burden, especially to upcoming anxiety, among the general population in Germany.

### 4.1 Time spent thinking about COVID‐19

First, we found that the participants spend a tremendous amount of time (285 min on average per day) thinking about COVID‐19‐related aspects during the day. If we compare this to the time amount of worrying healthy people usually show with a range between 28 and 55 min (Dupuy, Beaudoin, Rhéaume, Ladouceur, & Dugas, 2001; Verkuil, Brosschot, Gebhardt, & Thayer, 2010), this clearly exceeds the “normal” time period. On the one hand, addressing emerging problems by “constructive thinking” by finding solutions or gathering new important information for decision‐making may help coping with difficult situations (Drach‐Zahavy & Somech, 2002). On the other hand, ruminating as a “repetitive, prolonged, and recurrent negative thinking” is a vulnerability factor for anxiety, depression, and other mental disorders as well as raising physiological stress (Watkins & Roberts, 2020; Whisman, Du Pont, & Butterworth, 2020). Thus, our results underline the need of officially promoting a careful monitoring and regulation of the personal amount of time spent with thoughts about COVID‐19 in everyday life.

### 4.2 Risk perception of getting infected with COVID‐19

Second, we found that the risk perception of getting infected with COVID‐19 in the next 4 weeks was very high. These data show that as expected, the fear of becoming infected with COVID‐19 is very prevalent in the general population. Even in a time where the prevalence of COVID‐19 infections seems difficult to estimate, the risk rating of being infected within the next 4 weeks seems to be higher than the expected number of infections in 4 weeks. An infection probability of 40% within the next 4 weeks (the median) would mean over 30 million of infected people in Germany by beginning of May which seems rather unlikely when the current development is taken into account (Robert Koch Institut, 2020).

### 4.3 Anxiety regarding COVID‐19

Our data show, that overall, about half of the participants stated to be anxious about the consequences of the COVID‐19 pandemic on their life. Fears regarding COVID‐19 targeted more on social than on personal aspects. Besides the fear of general consequences, most fear was expressed with respect to consequences for the health of relatives as well as concerning the social consequences of the pandemic. Social consequences caused more concerns than economic ones. This result goes in line with other research showing that social support is very important for coping with adverse life events and reduces hopelessness (Tham, Ibrahim, Hunt, Kapur, & Gooding, 2020).

In the current situation, fears regarding the COVID‐19 pandemic have to be seen as normal consequences in an exceptional situation rather than as pathologic reactions (Petzold, Plag, & Ströhle, 2020a, 2020b). Differentiation, what amount of fear seems to be realistic and what is exaggerated, is almost impossible to draw. To get a picture of the percentage of people that develop a level of anxiety regarding COVID‐19 that itself leads to constraints in daily life, we asked the participants whether they thought that their anxiety is exaggerated and whether this led to limitations in their daily life. About 17% of the sample rated their level of anxiety as exaggerated, and about 25% of the sample stated that the anxiety itself would result in limitations in their daily life. These first data show that there is a relevant percentage of the general population, in which the anxiety regarding the COVID‐19 pandemic leads to significant burden in daily life. These proportions are comparable with findings from China during the initial COVID‐19 outbreak (Wang, Pan, Wan, Tan, Xu, Ho, et al., 2020), where more than half of the participants reported a moderate‐to‐severe psychological impact of the COVID‐19 pandemic on themselves, while about 17% of reported moderate‐to‐severe depressive symptoms and nearly 30% reported moderate‐to‐severe anxiety symptoms.

### 4.4 Risk and protective factors

Interestingly, personal experiences with COVID‐19 were not strongly connected to COVID‐19 anxiety. This could mean that psychological and social determinants may have a larger influence on anxiety in that early phase of the pandemic than immediate experiences with this virus itself. This is undermined by our finding that self‐efficacy (meaning a person’s believe in his or her own ability to master situations or show a certain behavior) showed essential significant negative correlations with COVID‐19 anxiety. Furthermore, the acceptance of anxiety and negative emotions, social support, and the knowledge, where to get treatment, if needed, were negatively associated with different aspects of COVID‐19 anxiety. This is in line with the recommendations on how to reduce the psychological distress in the pandemic (Inter‐Agency Standing Committee, 2020; International Federation of Red Cross & Red Crescent Societies, 2020; World Health Organization, 2020). For correlational analyses only allowing noncausal assumptions, we cannot determine the direction of these effects. Further longitudinal studies can give more information on causal relationships. For the factor of the acceptance of negative emotions, previous research that showed a negative circle of fear and suppression of anxiety during the Zika outbreak in Canada (Dillard, Yang, & Li, 2018) supports the hypothesis that the suppression of negative emotions might have an influence on future anxiety and negative emotions.

Low self‐efficacy has been shown to be connected with higher anxiety (Bandura, 1988; Muris, 2002). Our results make the assumption reasonable that self‐efficacy could be a protective factor also against pandemic‐driven anxiety and future longitudinal studies should test this assumption.

The result that working in a medical context is associated with more anxiety regarding the COVID‐19 pandemic is in line with findings from a recent study from hospitals in Singapore and India that showed high proportions of physical and psychological strain in healthcare workers (Chew et al., 2020). A further comparison of different professions in the healthcare sector would be interesting—as for example in a study in Singapore nonmedical healthcare workers (e.g., pharmacists, technicians) reported more psychological strain than medical personnel (Tan, Chew, et al., 2020; Tan, Hao, et al., 2020).

These results are of a high practical value as they empirically underpin the recommendations on the reduction of psychological distress in the current pandemic that are given by international organizations (Inter‐Agency Standing Committee, 2020; International Federation of Red Cross & Red Crescent Societies, 2020; World Health Organization, 2020) and show that the acceptance of anxiety and negative emotions, social contacts, self‐efficacy, and to know where to get medical treatment are important factors associated with reduced psychological burden. We also found evidence that supports the recommendation of maintaining a healthy lifestyle and to avoid suppression of negative emotions.

### 4.5 Depressive and anxiety symptoms

In our sample, the average PHQ‐4 Score was with a mean of 4.15 higher than the PHQ‐4 Score that has been reported by previous research in the general population of 1.76 (Löwe et al., 2010). With all given precautions, this could show that in the current situation there is an increase in depressive and anxiety symptoms in the German general population. Due to the nature of the study, this cannot be interpreted as a robust and reliable research result and should be merely seen as an empirical fundament to build hypotheses in this direction. If elevated levels of anxiety and depression turn out reliable and robust in other studies and especially in the longitudinal course, appropriate interventions should be established to reduce psychological strain—for example, cognitive behavioral therapy (Ho, 2020). In a first longitudinal study from China (Wang, Pan, Wan, Tan, Xu, McIntyre, et al., 2020), a statistically significant but not clinically relevant reduction in PTSD symptoms as a result of the COVID‐19 pandemic was found from end of January to end of February 2020. At the same time, there were no significant changes regarding anxiety, depression, and stress. Furthermore, the study identified protective factors such as confidence in doctors and satisfaction with health information, risk perception and outcome expectation (perceived survival likelihood), and personal precautionary measures (Wang, Pan, Wan, Tan, Xu, McIntyre, et al., 2020).

### 4.6 Gender effects

In our sample, women showed higher scores of COVID‐19 anxiety, more time of thinking about COVID‐19 per day, as well as more depressive symptoms than men. This is in line with the results of other studies regarding the psychosocial distress caused by the COVID‐19 pandemic (Qiu et al., 2020; Wang, Pan, Wan, Tan, Xu, Ho, et al., 2020). Up to now, it is not possible to draw conclusions if this is something specific to the COVID‐19 pandemic as higher values of anxiety and depression are reported in women in general (Salk, Hyde, & Abramson, 2017).

### 4.7 Strengths and limitations

Our study represents the first study that assesses psychological distress, anxiety, and depression as well as risk and protective factors in the current COVID‐19 pandemic in Germany. We started recruitment quite early so we assessed our participants still in a situation where case numbers were rising exponentially and media coverage was really large. This allows to study the psychological consequences at an early stage of the pandemic and lays a good basis for further longitudinal follow‐ups. With a sample size of over 6,000 participants, our sample is large enough to detect even small effects. Our sample was fully registered and approved by the local ethics committee.

Nevertheless, there are some limitations. We recruited our sample as convenience sample mainly through social media. This might have led to a sample bias. People who are familiar with or have easy access to social media might have been more likely to participate in our study, which might have led to a rather young sample. Furthermore, people who show higher levels of psychological distress and anxiety might be more likely to take part in a study like ours. This could have led to an overestimation of these factors in our sample. This strategy of recruitment does reduce the generalizability of our results which is shown by several differences between the demographics in our sample and the general population in Germany. The sample shows in comparison with the general population a much higher gender imbalance, a lower average age, and a higher percentage of persons working in a medical context (Bundesinstitut für Bevölkerungsforschung, 2020).

Our study is a cross‐sectional examination and does not allow any causal interferences. Our questionnaire was rather short, using simple scales, not all of them were validated. Therefore, all of the study results in general should rather be interpreted as first hints, which might be helpful for further studies as well as to empirically underpin existing recommendations on the reduction in psychological distress in the pandemic.

## 5 CONCLUSION

Our results suggest that in this early phase of the COVID‐19 pandemic with low percentages of diagnosed cases in our study population, we can already observe its fundamental impact on anxiety and psychological distress. This can be seen in about half of our sample stating fears regarding the consequences of the pandemic as well as in the high number of average hours of thinking about the pandemic per day. Regarding the role of protective and risk factors, our results suggest that there might be stronger links to psychological and social determinants and psychological distress as a result of the pandemic compared to personal experiences with COVID‐19 infections. The role of the recommended strategies to reduce psychological distress in the pandemic such as a healthy lifestyle, social support, acceptance of negative emotions, and avoidance of suppression and substance abuse is supported by our data.

## CONFLICT OF INTEREST

The authors declare that there is no conflict of interest.

## AUTHOR CONTRIBUTIONS

Moritz Bruno Petzold, Julia Große, and Antonia Bendau involved in literature research, designed the study, collected data, analyzed the data, interpreted the data, and wrote and revised the manuscript. Jens Plag involved in literature research and ethics committee communication, designed the study, and revised the manuscript. Lena Pyrkosch involved in literature research and data protection committee communication, designed the study, and revised the manuscript. Lea Mascarell Maricic involved in literature research, designed the study, translated the language, and revised the manuscript. Felix Betzler, Janina Rogoll, and Andreas Ströhle involved in literature research, designed the study, and revised the manuscript.

## ETHICAL STANDARDS

The authors assert that all procedures contributing to this work comply with the ethical standards of the relevant national and institutional committees on human experimentation and with the Helsinki Declaration of 1975, as revised in 2008.

### Peer Review

The peer review history for this article is available at https://publons.com/publon/10.1002/brb3.1745.

## Data Availability

The data that support the findings of this study are available from the corresponding author upon reasonable request.
